# Understanding the Swelling Behavior of Polysaccharide-Based Hydrogels through a Kinetic Modeling

**DOI:** 10.1021/acspolymersau.5c00208

**Published:** 2026-02-19

**Authors:** Vinicius Duarte Machado, Michele Karoline Lima-Tenório, Ernandes Taveira Tenório-Neto

**Affiliations:** Laboratory of Multifunctional Polymeric Materials (LMPM), Department of Chemistry, State University of Ponta Grossa (UEPG), Av. General Carlos Cavalcanti, 4748, Ponta Grossa, Paraná CEP: 84030-900, Brazil

**Keywords:** mathematical modeling, overshoot, hydrogel, mechanism, swelling

## Abstract

Hydrogels are versatile polymeric materials widely used in various applications, including drug delivery, agriculture, and environmental technologies. Their performance and applicability are mainly governed by the swelling behavior. As a result, an accurate description of swelling kinetics is crucial for understanding the transport mechanisms that guide the hydrogel design. However, the power-law model fails to describe the full swelling profile and transient phenomena, such as, nonmonotonic swelling. In this work, we propose a physical model based on a kinetic interpretation, which is capable of describing the entire swelling profile of hydrogels. The model is derived from fundamental transport concepts, and incorporates both Fickian diffusion and macromolecular relaxation within a unified framework. Importantly, several classical swelling equations—including the power law, first-order kinetic, Peppas–Sahlin, and Higuchi models—are shown to emerge as particular cases of the proposed formulation. The model was validated using experimental swelling data of chemically cross-linked polysaccharide-based hydrogels with different compositions. The proposed equation accurately fitted the swelling curves, including the overshooting behavior, with high correlation coefficients. The model quantified the contribution of Fickian diffusion and macromolecular relaxation in the swelling mechanism. The proposed model offers a simple and comprehensive tool for analyzing hydrogel swelling kinetics. By describing the full swelling process with only three physical parameters, it enables improved mechanistic interpretation, providing valuable guidance for the rational design of hydrogels with tailored swelling and absorption properties.

## Graphical abstract

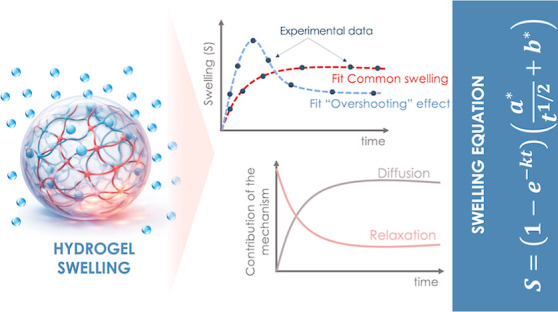

## 1 Introduction

Hydrogels (HGs) are a class of soft materials composed of three-dimensional polymeric networks capable of absorbing and retaining large amounts of water or biological fluids.^1^ Due to this remarkable swelling ability, the HGs have attracted continuous attention over the last decades. They have been explored in a wide range of applications, including controlled drug delivery systems,^2^ tissue engineering,^3^ sensors,^4^ agriculture,^5^ environmental remediation,^6^ and food technology.^7^ Their versatility arises from the possibility of tailoring chemical composition, cross-linking density, network architecture, and functional groups, allowing fine control over both structural and transport properties.

In this context, the swelling behavior of HGs is a key property that determines material performance and suitability for applications. For example, in drug delivery systems, the swelling controls drug diffusion and release rates,^7^ while in the agricultural or soil-conditioning applications, it determines water retention capacity and nutrient availability.^8^ Consequently, an accurate description of the swelling kinetics has long been recognized as a central challenge. Beyond equilibrium swelling, the time-dependent swelling profile offers valuable insights into the transport mechanisms. These mechanisms involve a complex interplay between solvent diffusion and the relaxation of the polymeric network.

Several mathematical descriptions have been proposed to represent swelling kinetics. It is commonly accepted that the mechanism is governed by at least two coupled processes: (i) solvent diffusion into the matrix and (ii) time-dependent rearrangement (relaxation) of the polymer network. Berens and Hopfenberg separated these contributions by treating them as distinct terms in glassy polymers.^9^

Early attempts to model swelling kinetics were largely inspired by Fickian diffusion concepts, assuming that solvent transport through the polymer matrix is the rate-determining step.^10^ Classical diffusion-based approaches were successfully applied to thin films and lightly cross-linked systems. However, they fail to describe the full swelling behavior of many polymer networks. A significant advancement in this field was the development of semiempirical kinetic models that explicitly incorporate non-Fickian transport mechanisms. Among these models, the power-law expression proposed by Peppas and colleagues is the most widely utilized tool for analyzing the mechanism of swelling.^1,11^ In its classical form, the power law relates the fractional uptake to time through the expression

1 $$S=kt^{n}$$

where *S* is the swelling degree, *k* is a proportionality constant, and *n* is the exponent that provides information about the transport mechanism, distinguishing between Fickian diffusion, case-II transport, and anomalous behavior.^12^ This approach has been extensively applied to polymeric hydrogels due to its simplicity and the apparent physical meaning of its parameters.

Further refinements led to models that explicitly separate the contributions of diffusion and polymeric relaxation. For example, Peppas and Sinclair derived an expression describing the penetrant front position as the sum of a diffusive term, α*t* ^1/2^, and a relaxational term, β*t*.^13^ Later, Peppas and Sahlin introduced similar additive terms to account for both mechanisms, providing a more detailed interpretation of swelling and release kinetics in complex systems.^14^ This formulation has been particularly useful for systems in which neither diffusion nor relaxation alone dominates the process. Nevertheless, despite their conceptual appeal and widespread acceptance, power-law-based approaches share an intrinsic limitation: they are valid only for the initial stages of swelling, typically restricted to the first 60% of the total uptake. As a result, they fail to describe the complete swelling profile, especially at longer times. This limitation is not merely mathematical but has direct physical implications, since restricting the analysis to the initial swelling stage may obscure the real contributions of diffusion and macromolecular relaxation.

In addition to these approaches, classic theories based on thermodynamics, the Flory–Rehner formalism, and rubber elasticity concepts have been adapted to hydrogel systems. These theories provide valuable insights into swelling at equilibrium.^12,15,16^ Others sophisticated approaches include first- and second-order kinetic models,^17^ molecular dynamics simulations,^18^ machine learning,^19^ and so forth.^20,21^

Despite these advances, many existing models are mathematically intensive, require numerous fitting parameters^22,23^ or they do not provide additional physical insight into the swelling mechanism.^10^ In addition, swelling kinetics may exhibit nonmonotonic behavior that conventional models may not capture. For example, several studies have reported a transient overshooting effect, where the hydrogel initially absorbs more solvent than it can sustain at equilibrium, followed by partial solvent release as the network relaxes.^24–26^ Such a fact illustrates the need for kinetic descriptions capable of capturing the entire swelling profile within a single and consistent theory.

Thus, despite the extensive literature and the undeniable success of existing models, a clear gap remains. The power-law and related semiempirical approaches may provide valuable mechanistic insight but are intrinsically limited to the quasi-linear region of swelling or its equilibrium. Moreover, none of these frameworks offers a simple, unified description capable of capturing the entire swelling process using a minimal set of physically meaningful parameters.

In this work, we address this gap by deriving a mathematical model that describes the complete swelling profile of hydrogels based on a kinetic approach. The proposed equation enables direct interpretation of the underlying transport mechanisms. Importantly, the model requires only three parameters: one associated with the swelling rate and two related to the relative contributions of diffusion and macromolecular relaxation. These parameters are obtained from the full swelling curve and retain the same physical interpretation throughout the entire process. In addition, we further demonstrate that several accepted swelling models, including the classical power law and related formulations, emerge as particular cases of the proposed equation under specific limiting conditions. By combining simplicity, physical meaning, and applicability, this model provides a robust tool for analyzing swelling kinetics in hydrogels.

## 2 Materials and Methods

### 2.1 Materials

Sodium hydroxide (NaOH, 99%) was acquired from Dinâmica. Sodium alginate from brown algae (Alg, *M*_W_ = 60 kDa, *M*/*G* ratio = 1.42), Pectin from citrus peel (*M*_w_ = 485 kDa, galacturonic acid ≥74%),^27^ glycidyl methacrylate (GMA) 97%, acrylic acid 99%, potassium persulfate ≥98%, *N,N*′-dimethyl acrylamide (DMAAm) 99% were purchased from Sigma-Aldrich. All reactants were used without further purification.

### 2.2 Chemical Modification of Polysaccharides with GMA

The chemical modification of polysaccharides with GMA has been extensively exploited by our research group.^28–30^ The chemical reaction between GMA and hydroxyl groups from carbohydrates occurs in a basic medium via an epoxide ring-opening mechanism. Under these conditions, two regioisomeric products may be formed: 3-methacryloyl-2-glyceryl ether of the polysaccharide and 3-methacryloyl-1-glyceryl ether of the polysaccharide (see the representative structures in Supporting Information).^28,31^ Regardless of the regioisomer, the functionalization introduces polymerizable vinyl groups into the polysaccharide backbone, enabling subsequent polymerization and the formation of chemically cross-linked hydrogels.

#### 2.2.1 Modification of Pectin

The chemical modification was carried out in a 250 mL three-neck round-bottom flask equipped with a reflux condenser. Four grams of the pectin were dissolved in 150 mL of distilled water under constant stirring. After complete dissolution, the pH of the solution was adjusted to 10 by the dropwise addition of 1.0 M NaOH. The temperature was then set to 60 °C, followed by the addition of 2.0 mL of GMA. The reaction was allowed to proceed for 24 h under constant stirring.^1^

#### 2.2.2 Modification of Sodium Alginate

Two grams of sodium alginate was dissolved in 60 mL of distilled water. The solution was added into a 100 mL round-bottom flask equipped with a reflux condenser. Then, the pH was adjusted to 4.0 by the addition of 0.1 M hydrochloric acid followed heating to 60 °C. Finally, 1.3 mL of GMA was added into the solution and the system was kept under constant stirring at 60 °C for 24 h.^32^

### 2.3 Synthesis of Polysaccharide-based Hydrogels

Before gelation, acrylic acid was neutralized with NaOH to obtain the corresponding acrylate salt. The use of the acrylate salt is preferred to prevent overheating during the polymerization step. Briefly, equimolar amounts of acrylic acid and NaOH were dissolved in acetone under continuous stirring. After 6 h, the resulting whitish suspension (acrylate salt) was vacuum-filtered and dried in a ventilated oven at 35 °C for 48 h.^1^

#### 2.3.1 Synthesis of Pectin-Based Hydrogels

Three different formulations were prepared by varying the amount of DMAAm. Initially, 5 mL of the modified-pectin solution was transferred to a 10 mL beaker. Subsequently, a predefined volume of DMAAm was added according to the formulation (Table 1). The system was then heated to 60 °C, followed by the addition of 0.02 g of sodium persulfate (initiator). The reaction took place for 30 min, and then the sample was left to cool to room temperature for another 30 min.

**Table 1:** Compositions of the Pectin-Based Hydrogels

sample name	DMAAm amount (mL)
H0.50	0.50
H0.75	0.75
H1.0	1.00

After synthesis, the hydrogels were dialyzed. Each formulation was placed in a beaker containing distilled water, which was periodically renewed every 6 h over a total period of 48 h to remove unreacted species and low-molecular-weight compounds. After that, the hydrogels were dried in a ventilated oven at 40 °C until reaching a constant mass.

#### 2.3.2 Synthesis of Alginate-Based Hydrogels

In an Eppendorf tube, 130 μL of the as-prepared alginate–GMA solution (33.4 g·L^–1^) was combined with the desired amount of sodium acrylate solution (500 g·L^–1^), DMAAm, and distilled water, according to the compositions listed in Table 2. The mixture was placed in a dry bath (INN Laboratories, model Mini 13) and the temperature was adjusted to 60 °C. Then, 24.4 μL of sodium persulfate solution (30 g·L^–1^) was added. The reaction took place for 15 min, and the sample was then allowed to cool to room temperature.

**Table 2:** Compositions of the Alginate-Based Hydrogels

sample name	sodium acrylate (μL)	DMAAm (μL)	H_2_O (μL)
A1	25.0	25.0	45.6
A2	37.5	18.8	39.4
A3	50.0	12.5	33.1

### 2.4 Fitting Data and Statistical Analysis

Statistical analysis of variance (ANOVA), Tukey tests, and correlations between the model and the experimental results were performed using Excel software. Model accuracy was validated by the least-squares method.

### 2.5 Swelling Measurements

The swelling kinetics of the samples were investigated by immersing a known amount of dried hydrogel into phosphate buffer solution (PBS, 0.1 M, pH 6.8) at 25 °C. Then, at specific time intervals, the hydrogels were withdrawn from the medium, their surface was wiped off carefully to remove the excess water droplets, and the samples were weighed. After, the hydrogels were returned to the solution. The data on swelling kinetics is shown as an average of three different experiments (*n = 3*). The swelling degree (*S*) is defined according to eq 2

2 $$S=\frac{w\left(t\right)-w_{o}}{w_{o}}$$

where *w*(*t*) is the weight of hydrogel measured at a specific time and *w*_o_ is the weight of dried hydrogel.

## 3 Results and Discussion

### 3.1 Mathematical Modeling

#### 3.1.1 Considerations on the Water Absorption

Hydrogels can be described as a cross-linked polymeric structure, which is hydrophilic, that can absorb some amount of water (or water-based fluids).^33^ Although such a structure is hydrophilic, it is worth mentioning here that the polymeric structure is not dissolved by the solvent.^34^

Depending on the nature of the polymer or the presence of other compounds, the water transport through polymeric chains is affected by pH, temperature, Ionic strength, magnetic field, and so forth.^28,35,36^ Here, to understand the process of water transport, such parameters will be considered constant.

According to Ganji et al., only a sorption process for polymer–solvent systems cannot describe the hydrogel swelling.^37^ Thus, the swelling mechanism is described by a contribution of Fickian diffusion and macromolecular relaxation.^1,13,38^ The current theoretical swelling models consider that both contributions are independent, expressing the change of hydrogel weight over time as a linear combination of them.^9,10^ Here, we propose that relaxation and diffusion contributions are intrinsically related to a single function that is associated with the kinetics of penetration of the solvent. For a better understanding of our statement, a simplified schema representing the swelling process is shown in Figure 1.

**Figure 1:**
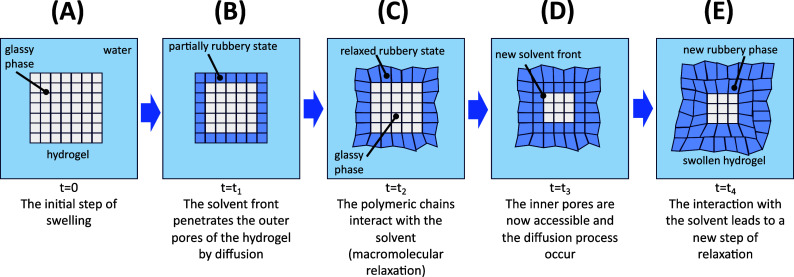
A simplified schema representing the steps involved in a swelling process.

A dry hydrogel is in its vitreous form (Figure 1A). In a liquid medium, the solvent front penetrates and fills the free volumes by diffusion. Most of the inner free volume is not accessible at this moment (Figure 1B). If the polymer–solvent interaction is favorable, there will be a relaxation, expanding the polymeric chains (Figure 1C). Then, the inner free volumes become accessible, which allows the solvent front to penetrate in the HG through a diffusion process (Figure 1D). After, there will be new interactions with the inner polymeric chains, by a relaxation process, enabling more inner free volumes (Figure 1E). This process repeats until the swelling equilibrium is reached.

Thus, we consider that both mechanisms are intrinsically related to each other. Thus, the evolution of the swelling process should be given as a product of two functions as follows

3 S=F(t)·G(t)

where, *F*(*t*) and *G*(*t*) are functions related to the kinetic diffusion of solvent and a function describing the expansion of the polymeric network, respectively.

The boundary conditions of eq 3 appear naturally. For example, in the initial stage of swelling (*t* = 0), there is no solvent penetration. Thus, *F*(0) must be equal to zero, leading to *S* = 0. On the other hand, when *t* → ∞, both *F*(∞) and *G*(∞) must become a constant, since the polymeric chains expanded until their maximum volume. In addition, if *G*(*t*) is a constant, this implies that the system does not expand (constant volume), although the system may absorb the solvent by diffusion.

Below, we will provide a detailed explanation of the derivation of the functions *F*(*t*) and *G*(*t*).

#### 3.1.2 The Function *F*(*t*)

The function *F*(*t*) is related to the speed of solvent penetration in the HG system, which occurs due to a concentration gradient. Thus, we can expect that *F*(*t*) should be proportional to the mass of absorbed water as a function of time. By the balance mass, the weight of the hydrogel at any time (*w*(*t*)) is given by

4 $$w\left(t\right)=w_{o}+w_{water}\left(t\right)$$

where *w*_water_(*t*) is the weight of absorbed water. When the hydrogel achieves the equilibrium *w*_water_(*t*) = $w_{water}^{\mathrm{Max}}$ , and eq 4 becomes constant: *w*_eq_ = *w*_o_ + $w_{water}^{\mathrm{Max}}$ .

Considering a system with constant volume, the rate of water that is absorbed by the hydrogel is directly proportional to the difference between maximum water uptake ( $w_{water}^{\mathrm{Max}}$ ) and *w*_water_(*t*) (unrealized water uptake)

5 $$\frac{dw_{water}\left(t\right)}{dt}\propto w_{water}^{\max}-w_{water}\left(t\right)$$

Then

6 $$\frac{dw_{water}\left(t\right)}{dt}=k\left[w_{water}^{\max}-w_{water}\left(t\right)\right]$$

where *k* is a kinetic constant related to the speed of solvent penetration into the system.

The solution of eq 6 is given below (see Supporting Information)­

7 $$w_{water}\left(t\right)=w_{water}^{\max}+Ae^{-kt}$$

where *A* is a constant of integration. Thus, the *w*(*t*) function will be given by

8 $$w\left(t\right)=w_{o}+w_{water}^{\max}+Ae^{-kt}$$

Finally, as the function *F*(*t*) is proportional to *w*(*t*), and that *w*_eq_ = *w*_o_ + $w_{water}^{\mathrm{Max}}$ , we obtained the eq 9

9 $$F\left(t\right)=\alpha w_{eq}\left(1-e^{-kt}\right)$$

where α is the proportionality constant.

#### 3.1.3 The Function *G*(*t*)

The *G*(*t*) function is related to the expansion of polymeric chains (or interaction with the solvent) and not necessarily to an increase in the mass. Such an expansion can be understood considering the movement of the cross-linking points (Figure 2).

**Figure 2:**
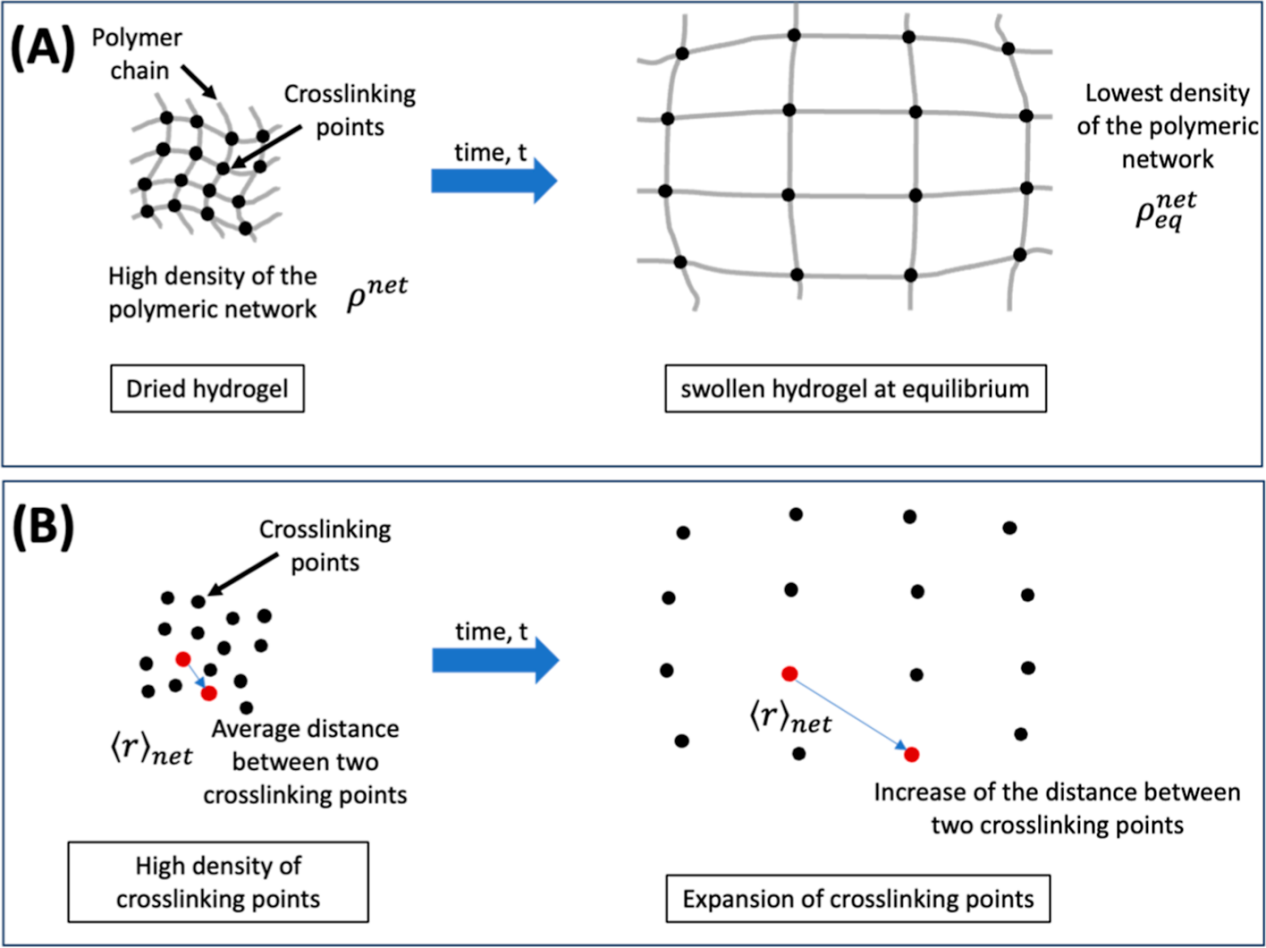
Representation of the polymeric chain’s expansion during swelling (A). Representation of the expansion of only the cross-linking points (B).

The network density of a hydrogel (ρ^net^) is defined as the ratio between the dry mass of the hydrogel (*w*_o_) and its volume (*V*) during swelling

10 $$\rho^{net}=\frac{w_{o}}{V}$$

As the hydrogel swells, the distance between its cross-linking points increases, resulting in a decrease in network density. At equilibrium, the ρ^net^ becomes $\rho_{eq}^{net}$ , which is the minimum density of the hydrogel network. We assume that the time dependence of ρ^net^ should be proportional to the rate of change of the distance between two cross-linking points with time (d⟨*r*⟩_net_/d*t*). In addition, the *G*(*t*) function is proportional to the ρ^net^.

Considering that each point moves randomly, the temporal dependence of the average distance between two cross-linking points (⟨*r*⟩_net_) can be modeled like a gas expansion through a random walk approach. The average distance traveled between two cross-linking points, ⟨*r*⟩_net_, is proportional to *t* ^1/2^(see Supporting Information).

Therefore, by deriving ⟨*r*⟩_net_ with respect to time, the rate of change will be proportional to *t* ^–1/2^. Thus, the ρ^net^ should be given by (see the derivation in Supporting Information)­

11 $$\rho^{net}=\frac{\beta }{t^{1/2}}+\rho_{eq}^{net}$$

where β is a constant. It is important to highlight here that the eq 11 can be verified experimentally. Finally, the *G*(*t*)­function should be given by

12 $$G\left(t\right)=\delta \left(\frac{\beta^{\prime }}{t^{1/2}}+\varepsilon \right)$$

where, β^′^, δ, and ε are proportional constants.

##### 3.1.3.1 Experimental Evaluation of the ρ^net^ and its Temporal Dependence

To evaluate the dependence of ρ^net^ as a function of time, three different pectin-based hydrogels were synthesized (Table 1). The main difference between samples is the amount of DMAAm, which increases the polarity and the chain density without introducing additional intermolecular interactions (e.g., hydrogen bonding, ionization). The sample was swollen in PBS to control the external environment. During swelling, at a certain time interval, the volume of each hydrogel was measured by Archimedes’ principle. All experiments were done in triplicate. The dependence of ρ^net^ with respect to time is shown in Figure 3.

**Figure 3:**
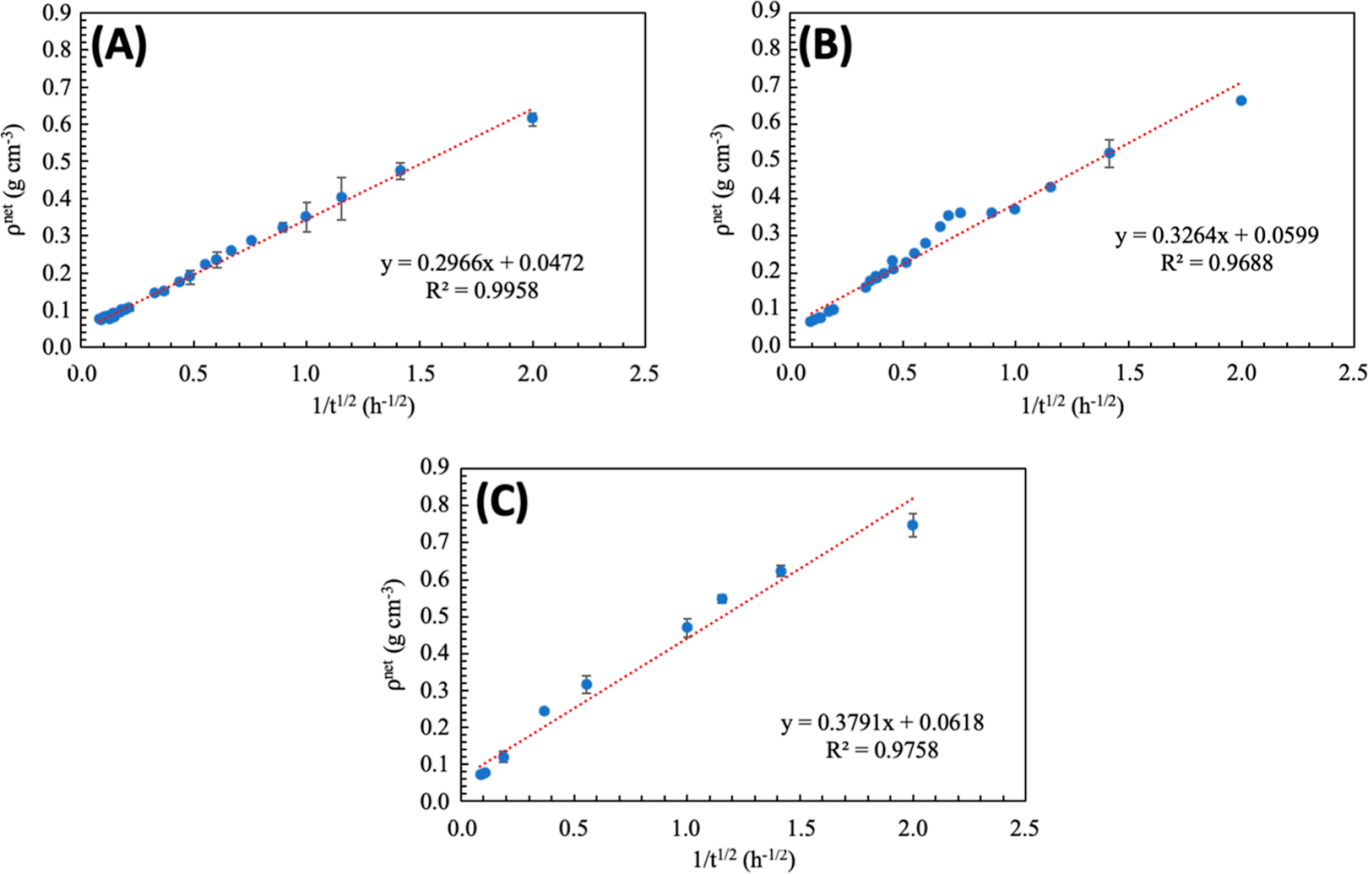
Results of measurements ρ^net^ as a function of *t* ^–1/2^ obtained for different hydrogel samples: HG0.5 (A), HG0.75 (B), and HG1.0 (C).

The linear dependence of ρ^net^ on *t* ^–1/2^ was observed for all samples, supporting the applicability of eq 10 to describe the hydrogel expansion behavior. This finding indicates that the assumptions of our model are consistent with the experimental results. In addition, the form of eq 10 is similar to the relationship that predict the evolution of mesh size of systems with lower volume fraction of polymer.^22^ Moreover, the agreement between *R* ^2^ and adjusted *R* ^2^ suggests that the parameters included in eq 10 are sufficient for capturing the physical mechanisms that govern the hydrogel expansion, and no additional variables are required to improve the model’s accuracy.

According to eq 11, the positive correlation between the slope (β) and the expansion ratio may provide an insight into the role of polymer–solvent interactions. The corresponding values are shown in Table 3.

**Table 3:** Values of Coefficients from eq 10 Obtained for Pectin-Based Hydrogels^a^

sample	β	$\rho_{eq}^{net}$	*R* ^2^	*R* ^2^-adjust
HG 0.50	0.2966 ± 0.0085^a^	0.0472 ± 0.0059^a^	0.9958	0.9957
HG 0.75	0.3264 ± 0.0259^a,b^	0.0599 ± 0.0189^a^	0.9688	0.9674
HG 1.0	0.3791 ± 0.0450^b^	0.0618 ± 0.0404^a^	0.9758	0.9731

a Different superscript letters indicate statistically significant differences between the means (Tuckey test 95% confidence level, *n* = 3).

Due to the hydrophilic nature of DMAAm, an increase in its concentration in the HG increases the polarity of the polymer network. This structural modification facilitates stronger interactions with the solvent, promoting a more pronounced network expansion. This interaction could be quantified by measuring the β parameter. Its values increased from 0.2966 to 0.3791 (for samples HG0.5 to HG1.0) as the DMAAm content increased, which aligns with this interpretation supporting the hypothesis that β are related to the intensity of polymer–solvent affinity.

Despite this trend, the equilibrium value $\rho_{eq}^{net}$ did not differ significantly among samples. Therefore, although the swelling kinetics are sensitive to variations in DMAAm content, the final equilibrium state is governed by factors that remain relatively unchanged across formulations. These may include the effective cross-linking density or network architecture, which may impose similar constraints on maximum solvent uptake. Thus, changes in DMAAm concentration appear to influence the swelling pathway more strongly than the final equilibrium configuration.

#### 3.1.4 The Swelling Equation

Based on the exposed above, and by combining *F*(*t*) with *G*(*t*) (eqs 9 and 12), respectively) we could obtain the swelling equation as a function of time

13 $$S=\alpha w_{eq}\left(1-e^{-kt}\right)\delta \left(\frac{\beta^{\prime }}{t^{1/2}}+\varepsilon \right)$$

To simplify the eq 13, all the constants α, *w*_eq_, δ, β^′^, and ε were grouped as only two parameters a* and b* characteristic of the material

14 $$S=\left(1-e^{-kt}\right)\left(\frac{a^{*}}{t^{1/2}}+b^{*}\right)$$

where a* = α*w*_eq_δβ^′^, and b* = α*w*_eq_δε.

Thus, the swelling eq (eq 14), have only three parameters: *k* which is related to the speed of solvent penetration and the a* and b* which is network parameter related to the absorption mechanism, as will be demonstrated below.

### 3.2 The Generality of the Swelling Equation

In the literature, different swelling models have been proposed.^9,12,13,38^ As shown below, we will demonstrate that these equations are a special case of eq 14.

#### 3.2.1 The Power-Law Equation

In the earlier stage of solvent absorption (linear portion), the exponential term of eq 14 can be expanded by a Taylor series, *e* ^–*kt*^ ≈ 1 – *kt*, leading to

15 $$S\approx \left(1-1+kt\right)\left(\frac{a^{*}}{t^{1/2}}+b^{*}\right)$$

which can be simplified to

16 $$S\approx ka^{*}t^{1/2}+kb^{*}t$$

Such a relationship, is very similar to the power-law equation commonly used to investigate the swelling mechanisms on the first 60% of absorption (linear portion).^39,40^ By the power-law equation, the mechanism can be determined with the value of the shape-dependent exponential coefficient *n*. Usually, when *n* is between 0.43 and 0.50, the swelling mechanism is termed Fickian diffusion. On the other hand, for *n* between 0.85 and 1.0 the mechanism is controlled by macromolecular relaxation. For intermediate values the mechanism is considered anomalous, where there is the contribution of both diffusion and macromolecular relaxation.^41^

Similar results can be obtained from eq 16. For example, in a limit case, when a* → 0, *S* ≈ kb*t, and the exponent trend to 1, indicating that the absorption mechanism will be macromolecular relaxation. When, b* → 0, *S* ≈ ka*t ^1/2^, the exponent trend to 0.5, indicating that absorption mechanism follows a Fickian diffusion. Moreover, when a* and b* shows the same order, the mechanism will be termed as anomalous transport. Thus, we could conclude that a* and b* are the polymeric parameters which defines the mechanism.

A similar expression of eq 16 was derived by Peppas and Sinclair, which described the penetrant front position as the sum of a diffusive term, α*t* ^1/2^, and a relaxational term, β*t*.^13^ Later, this relationship was derived by Peppas and Sahlin for swellable-controlled release systems.^14^ In that work, the Fickian diffusion was expressed as a function of *t* ^*m*^ and while the relaxational contribution as a function of *t* ^2*m*^. Here, we demonstrated that the same relationship can be applied to also for investigating the swelling mechanism independently of the hydrogel shape.

#### 3.2.2 Expanding More Terms of Taylor Series

A short-time approximation method is another equation often used to estimate the diffusion coefficient (*D*) of spherical or cylindrical hydrogels as follows^42^

17 $$\frac{S}{S_{eq}}=4{\left(\frac{Dt}{\pi r^{2}}\right)}^{1/2}-\pi \left(\frac{Dt}{\pi r^{2}}\right)-\frac{\pi }{3}{\left(\frac{Dt}{\pi r^{2}}\right)}^{3/2}$$

the diffusion coefficient is estimated by *D* = π*r* ^2^(*k*/4)^1/*n*^, where *n* and *k* are respectively the exponential parameter and the constant of the power-law equation.^20,42,43^

A similar relationship can be derived from eq 14, considering that between the earlier and the middle swelling process, the exponential part of that can be expanded by the Taylor series with more terms:

18 $$S\approx \left[kt-\frac{{\left(kt\right)}^{2}}{2!}+\frac{{\left(kt\right)}^{3}}{3!}-\frac{{\left(kt\right)}^{4}}{4!}+...\right]\left(\frac{a^{*}}{t^{1/2}}+b^{*}\right)$$

which can be rewritten as

19 $$S\approx ka^{*}t^{1/2}+kb^{*}t-\frac{k^{2}a^{*}t^{3/2}}{2!}-\frac{k^{2}b^{*}t^{2}}{2!}+\frac{k^{3}a^{*}t^{5/2}}{3!}-\frac{k^{3}b^{*}t^{3}}{3!}\cdot \cdot \cdot$$

this equation shows the dependence of swelling with time raised to a power of *n*/2, with *n* = 1, 2, 3, etc.

##### 3.2.2.1 The Relationship of *a* ^*^ and *b* ^*^ with the Swelling Mechanism

Another interesting relationship can be obtained from eq 19, when the infinite series is grouped in terms of a* and b*

$$S=\underset{f}{\underset{︸}{a^{*}\;\left(\overset{\infty }{\underset{n=1}{\sum }}\frac{{\left(-1\right)}^{n+1}k^{n}t^{n-1/2}}{n!}\right)}}+\underset{r}{\underset{︸}{b^{*}\;\left(\overset{\infty }{\underset{n=1}{\sum }}\frac{{\left(-1\right)}^{n+1}k^{n}t^{n}}{n!}\right)}}$$ 20

The eq 20 represents a linear superposition of both mechanisms, the Fickian diffusion (*f*) (first term), and the macromolecular relaxation (*r*) (second term) as proposed in literature (linear combination).^9,10^ Thus, by using the eq 20, we can estimate the contribution of both mechanisms in the swelling measurements, by the following relation

21 $$CF=\frac{f}{f+r}$$

where *CF* is the relative contribution of Fickian diffusion to the mechanism. Therefore, *CF* can be estimated by (see Supporting Information)­

22 $$CF=\frac{1}{1+\frac{b^{*}t^{1/2}}{a^{*}}}$$

while the contribution of macromolecular relaxation (*CR*) is given by *CR* = 1–*CF*. For hydrogels applied in drug delivery systems, a similar equation was proposed by Peppas and Sahlin.^14^ Here, we demonstrated that such a model is a special case of our equation, and this relationship can also be used for modeling the swelling.

The form of eq 22 indicates that the parameter *k* is not relevant to determine the mechanism. By the definition modeling, *k* is related only to the speed of solvent absorption. Another important finding is that macromolecular relaxation or even Fickian diffusion will be not obtained only in a limit case (a* → 0 or b* → 0, respectively), but with the relationship between the parameters a* and b*. For example, simulations using eq 21 indicate that the macromolecular relaxation mechanism will occur when b* is c.a. ten times higher than a* (Figure 4A). On the other hand, when a* is higher than a hundred times b*, the mechanism will be trend to a Fickian diffusion (Figure 4B). This result is in agreement with the well-established power law equation, where the value of diffusional exponent *n* is shape-dependent, and does not have a defined value for indicating the mechanism. For Fickian diffusion, for example, if the shape of the hydrogel is a thin film, cylinder or sphere the value of *n* of power law equation will be between 0.50, 0.45, and 0.43, respectively. Such a result is related to a minimum contribution from macromolecular relaxation over the mechanism, which biases the result from *t* ^1/2^ downward.

**Figure 4:**
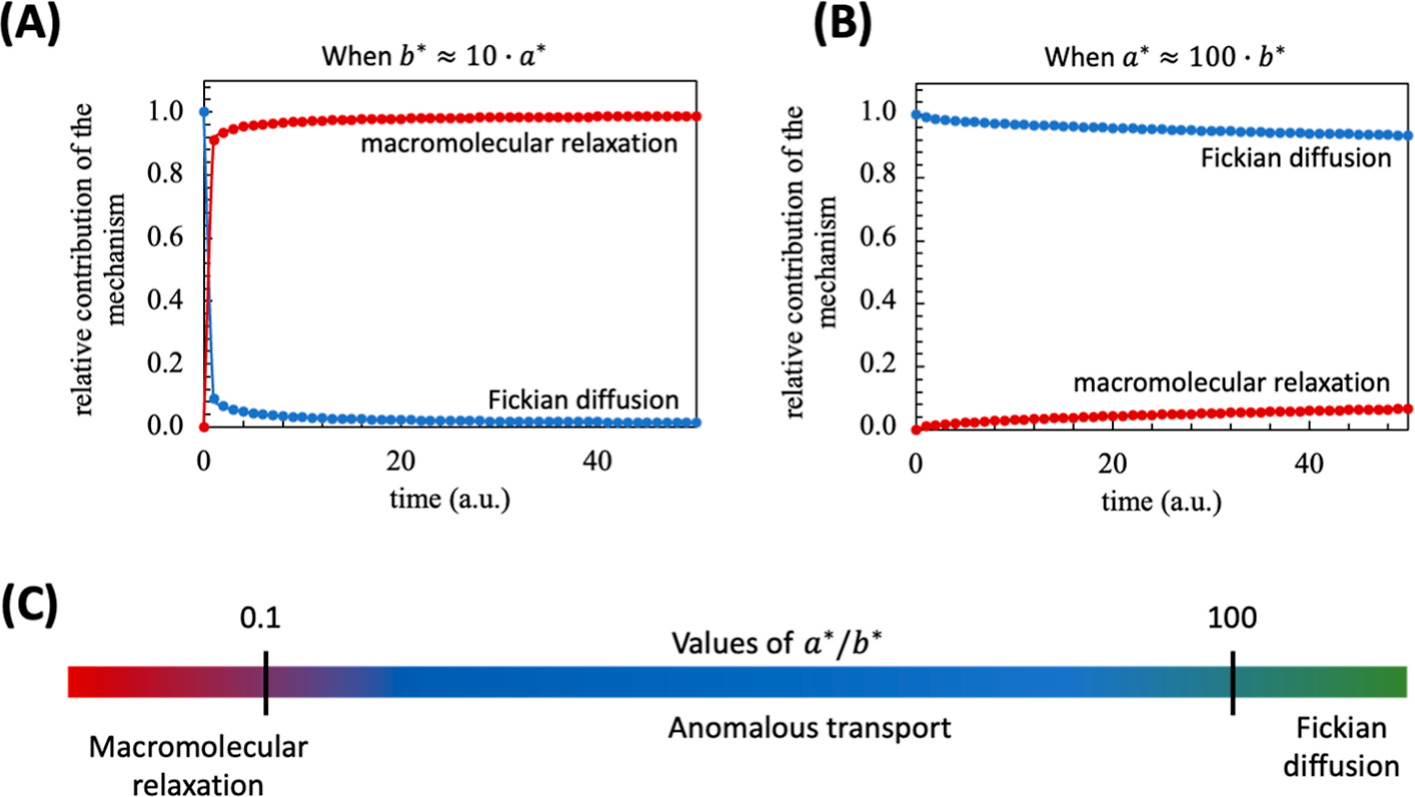
Simulations of mechanism contribution during a swelling for different values of a* and b*. The predominance of macromolecular relaxation (A), the predominance of Fickian diffusion (B), and a proposed scale to determine the swelling mechanism (C). Simulations obtained for *k* = 0.1.

Based on the above, in Figure 4C, a scale is proposed for determining the mechanism as a function of a*/b*. Note that both mechanisms occur simultaneously, with no defined limit, neither relaxation nor diffusion, except in the limit case. However, based on the value established between a*/b*, the contribution of the respective mechanism will be close to 100%.

#### 3.2.3 First-Order Kinetics

Based on the second Fick’s law, Berens and Hopfenberg described the relaxation process of a swelling mechanism as being first order as follows^9^

23 $$S=S_{\infty }\left(1-e^{-kt}\right)$$

Such an expression is also a special case of our model. For a relaxation-controlled mechanism (b*≫a*), the second term of eq 14 becomes constant, which leads to

24 $$S\approx b^{*}\left(1-e^{-kt}\right)$$

which are similar to the first-order kinetics. Here, it is important to point out that the b* parameter is defined as a product between different constants (b* = α*w*_eq_δε), as mentioned in Section 3.1.4. Thus, besides b* and *S*_∞_ has different meanings, but they are directly proportional.

### 3.3 Fitting Experimental Results

#### 3.3.1 Pectin-Based Hydrogels

The experimental results of swelling for samples HG0.50 to HG1.0 were fitted by the swelling equation and by the power law for comparison (Figure 5). These formulations were chosen to investigate the effect of increasing the DMAAm on the hydrogel formulation. In the literature, it is well-known that DMAAm acts as a spacer agent, increasing the chain polarity.

**Figure 5:**
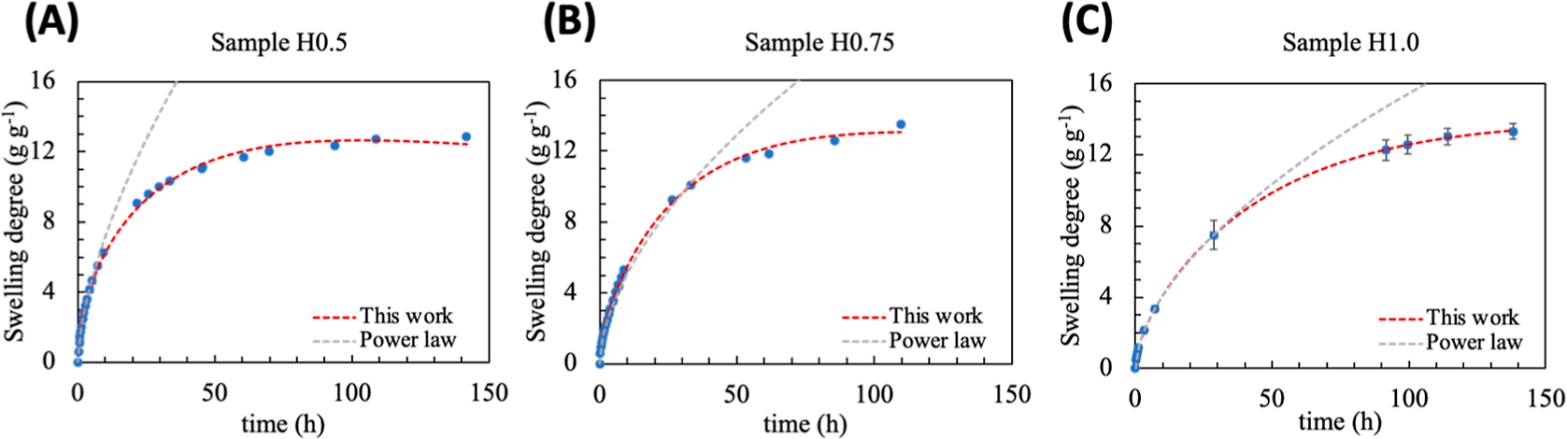
Time-dependent swelling curves of pectin-based hydrogels containing different amount of DMAAm: 0.50 mL (A), 0.75 mL (B), and 1.00 mL (C). Dashed line: modeling the experimental result by using the swelling equation (red line), and the power law (gray line).

The power law equation can adjust the experimental results only in the linear portion (approximately the first 60% of absorption). On the other hand, the swelling equation could describe the overall swelling with a coefficient of determination (*R* ^2^) higher than 0.99.

All samples showed a similar swelling equilibrium, ranging from 12 to 13 g·g^–1^. This result agrees with the observed in Section 3.1.3.1: even increasing the solvent affinity, the $\rho_{eq}^{net}$ did not differ significantly among samples. Thus, it is expected that the HGs absorb the same amount of solvent in equilibrium.

An investigation using the power law model indicates that the transport mechanism for all samples is anomalous, and the exponent *n* did not statistically differ between them (Table 4).

**Table 4:** Swelling Parameters of Pectin Hydrogels Obtained by the Power Law and the Swelling Equation^a^

	power law	swelling equation				
sample	*n*	transport mechanism	*k* (h^–1^)	a*/b*	*R* ^2^	transport mechanism
H0.50	0.63 ± 0.03^a^	anomalous	0.0211 ± 0.0066^a^	20.79 ± 16.41^a^	0.9977	anomalous
H0.75	0.58 ± 0.02^a^	anomalous	0.0239 ± 0.0010^a^	5.84 ± 0.52^a^	0.9983	anomalous
H1.00	0.58 ± 0.02^a^	anomalous	0.0148 ± 0.0054^a^	7.13 ± 4.23^a^	0.9999	anomalous

a Different superscript letters indicate statistically significant differences between the means (Tuckey test 95% confidence level, *n* = 3).

The swelling equation also indicated that the transport mechanism is anomalous, showing the same trend in changing the a*/b* ratio. However, our model could provide more information regarding the mechanism. Although the *k* did not differ statistically between samples, its average value seems to achieve a maximum value for sample H0.75.

In addition, the mechanism can be better understood by the analysis of a*/b* ratio. For example, the sample H0.50 showed a*/b* value equal to 20.79 which indicated anomalous transport with a certain Fickian contribution (see Figure 6A). As the amount of DMAAm increases, the Fickian contribution becomes less predominant. Due to the interaction between the polar chains (from DMAAm) with the solvent, there is a change in the predominant mechanism, which tends to macromolecular relaxation in long times (Figure 6B,C).

**Figure 6:**
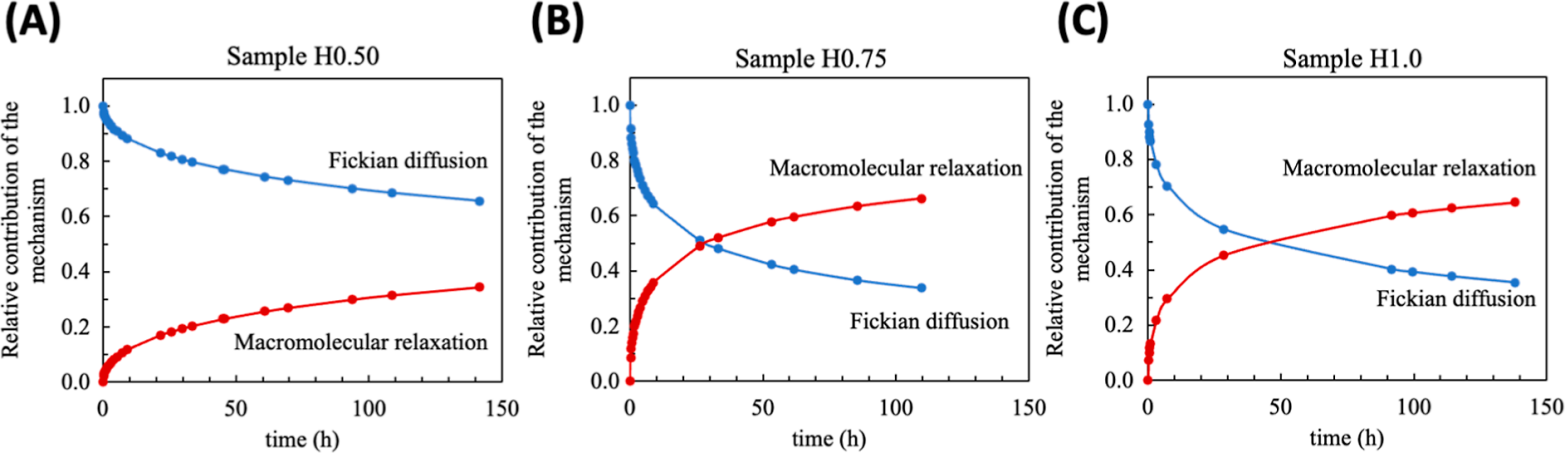
Contribution of macromolecular relaxation and Fickian diffusion for the samples HG0.50 (A), HG0.75 (B), and HG1.00 (C).

#### 3.3.2 Alginate-Based Hydrogels

The hydrogel compositions were deliberately designed to evaluate the effect of increasing sodium acrylate (SA) content on the swelling mechanism, while keeping both the total hydrogel volume and the alginate concentration constant. In this formulation set, the progressive increase in SA is accompanied by a proportional reduction in DMAAm, allowing the impact of introducing ionizable groups into the polymer network to be assessed without changing the overall polymer content.

Figure 7 shows the swelling profiles from sample A1 (lower SA content) to A3 (highest SA content). A clear formulation-dependent effect on the equilibrium swelling degree is observed. Specifically, increasing the SA content leads to a progressive increase in the equilibrium swelling degree, with values of approximately 24 for the A1 sample, 25 for the A2, and close to 30 for the A3. This behavior is consistent with the higher concentration of negatively charged acrylate groups at neutral pH, which increases osmotic pressure within the hydrogel, enhancing the fluid uptake by the polymer network.^44^

**Figure 7:**
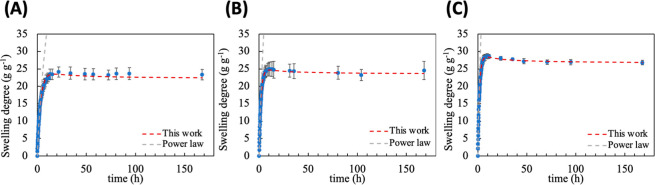
Time-dependent swelling curves of alginate-based hydrogels containing substituting the amount of DMAAm by SA: Sample A1 (A), sample A2 (B), and sample A3 (C). Dashed line: modeling the experimental result by using the swelling equation (red line), and the power law (gray line).

The power-law model could adjust only the initial (linear) region of the swelling process (up to 60%). In contrast, the swelling equation describes the entire swelling profile for all samples, yielding coefficients of determination higher than 0.99, as shown in Table 5.

**Table 5:** Swelling Parameters of Alginate Hydrogels Obtained by the Power Law and the Swelling Equation^a^

	power law	swelling equation				
sample	*n*	transport mechanism	*k* (h^–1^)	a*/b*	*R* ^2^	transport mechanism
A1	0.70 ± 0.06^a^	anomalous	0.2802 ± 0.0333^a^	0.35 ± 0.14^a^	0.9962	macromolecular relaxation
A2	0.74 ± 0.04^a^	anomalous	0.5080 ± 0.1215^b^	0.22 ± 0.05^a^	0.9990	macromolecular relaxation
A3	0.77 ± 0.07^a^	anomalous	0.5200 ± 0.0599^b^	0.27 ± 0.11^a^	0.9986	macromolecular relaxation

a Different superscript letters indicate statistically significant differences between the means (Tuckey test 95% confidence level, *n* = 3).

At early times (around 10 h), the samples A2 and A3 exhibited an overshooting effect, where the amount of absorbed fluid achieves a maximum followed by a gradual decrease until the swelling equilibrium. The occurrence of transient overshooting during swelling has been reported for several polymeric gel systems and is commonly associated with macromolecular relaxation phenomena.^45,46^ The earliest interpretation was proposed by Smith and Peppas, who observed a nonmonotonic swelling behavior in polystyrene gels swollen in cyclohexane. They attributed this effect to the delayed relaxation of the polymer chains relative to solvent diffusion.^24^ Yin et al. reported similar behavior in alginate-based hydrogels and explicitly associated the overshooting effect with the relaxation processes of macromolecular chains within the hydrogel network.^25^ Their results demonstrated that, in ionizable polysaccharide networks, the swelling kinetics are governed by the competition between fast solvent diffusion and slower chain relaxation, leading to a temporary overexpansion before equilibrium is reached. Kowalski and coauthors demonstrated that, in alginate-based hydrogels, the overshooting effect becomes more pronounced when the alginate content exceeds 4 wt %, indicating that increased polymer concentration enhances relaxation constraints within the network.^26^

The power-law model does not describe this behavior, whereas the proposed swelling equation captures it adequately, reinforcing its ability to represent the full swelling process.

The fit by using the power-law model resulted in diffusional exponents between 0.70 and 0.77, indicating anomalous transport for all formulations. On the other hand, the a*/b* ratios obtained from the swelling model range from 0.2 to 0.4, which are also characteristic of anomalous transport with a strong contribution from macromolecular relaxation. In both approaches, no significant changes in the swelling mechanism were observed as a function of composition. However, the *k* value increases significantly from A1 to A2, indicating that the highest sodium acrylate content accelerates the water uptake. In contrast, no significant change in *k* is observed between A2 and A3, suggesting that above a certain acrylate concentration, the swelling rate becomes limited by network relaxation rather than by electrostatic interactions.

The relative contributions of diffusion and macromolecular relaxation are shown in Figure 8. This approach provides an overview of the mechanism. At the early stage of swelling, there is a significant contribution from Fickian diffusion (c.a. 20%); thus, the power law equation led us to consider the transport mechanism as anomalous. However, the Fickian contribution decreases over time. As a result, the swelling initially proceeds via anomalous transport combining the diffusive and relaxational contributions and gradually becomes a relaxation-controlled regime. This result is consistent with the presence of ionizable groups (–COO^–^) and the overshooting behavior. Importantly, this transition in the swelling mechanism was not dependent on the sample composition.

**Figure 8:**
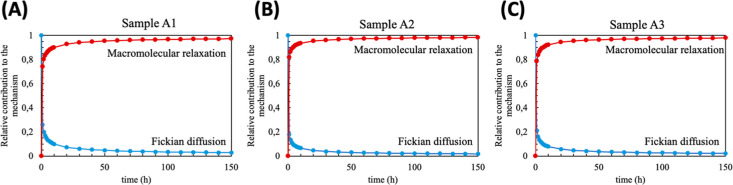
Contribution of macromolecular relaxation and Fickian diffusion for the samples A1 (A), A2 (B), and A3 (C).

These results demonstrate that the proposed swelling equation provides a consistent and physically meaningful description of the entire swelling process, capturing both kinetic and mechanistic features that are not accessible through conventional approaches.

## 4 Conclusions

In this work, a swelling model based on physical and kinetic principles was developed to describe the swelling behavior of hydrogels. The assumptions of the model are in good agreement with the experimental observations. This correspondence reinforces the physical meaning of the proposed formulation and its ability to capture essential structural features of polymer networks.

A major advantage of the proposed model is the possibility to describe the entire swelling profile, from the initial solvent uptake to the equilibrium state. This represents a significant improvement over commonly used approaches, such as the power-law model, which is limited to the linear region of swelling. Furthermore, it was demonstrated that several models emerge as particular cases of the proposed swelling equation. Classical models such as the power law, first-order kinetic models, Peppas–Sahlin, and Higuchi formulations can all be derived from the general expression, which also explained the dependence of swelling on time as a power series of *n*/2. This result provides a unifying framework that rationalizes the applicability and limitations of existing models within a single theoretical structure.

Another important contribution of the proposed model is its ability to quantitatively evaluate the relative contributions of Fickian diffusion and macromolecular relaxation throughout the entire swelling process. This feature makes the model a valuable tool for assessing hydrogel properties and for guiding the rational design of polymer networks. The model successfully predicts and describes the overshooting effect observed in certain hydrogel systems with results consistent to the observed in literature. Finally, the model also introduces a kinetic parameter directly associated with the rate of fluid absorption, which can be particularly useful for the development of hydrogels with fast swelling or rapid response characteristics. The model demonstrates its robustness and broad applicability, reinforcing its potential as a general tool for the analysis and interpretation of hydrogel swelling.

## Supplementary Material



## Notes

Schematic representation of chemical modification of polysaccharides; Integration of eq 5; A description of the random movement between two cross-linking points; Derivation of the temporal dependence of ρ^net^; Derivation of the relative contribution of Fickian diffusion to the mechanism (eq 21) (PDF)
