# Supplementary material for: Understanding the Swelling Behavior of Polysaccharide-Based Hydrogels through a Kinetic Modeling
Source: ACS Polym Au. 2026 Feb 19;6(2):634–44. doi: 10.1021/acspolymersau.5c00208 (PMC13067165; doi:10.1021/acspolymersau.5c00208)
Supplement: Supplementary file 1 [file lg5c00208_si_001.pdf]

Understanding the swelling behavior of polysaccharide-based hydrogels through a kinetic modelling

Vinicius Duarte Machado[a], Michele K. Lima-Tenório[a], Ernandes Taveira Tenório-Neto[a,*]

a. Laboratory of Multifunctional Polymeric Materials (LMPM), Department of Chemistry, State University of Ponta Grossa (UEPG), Av. General Carlos Cavalcanti, 4748, Ponta Grossa, Paraná, Brazil, CEP: 84030-900.

* Corresponding author: ernandes.tenorio@uepg.br

Supporting information

S1. Chemical modification of polysaccharides

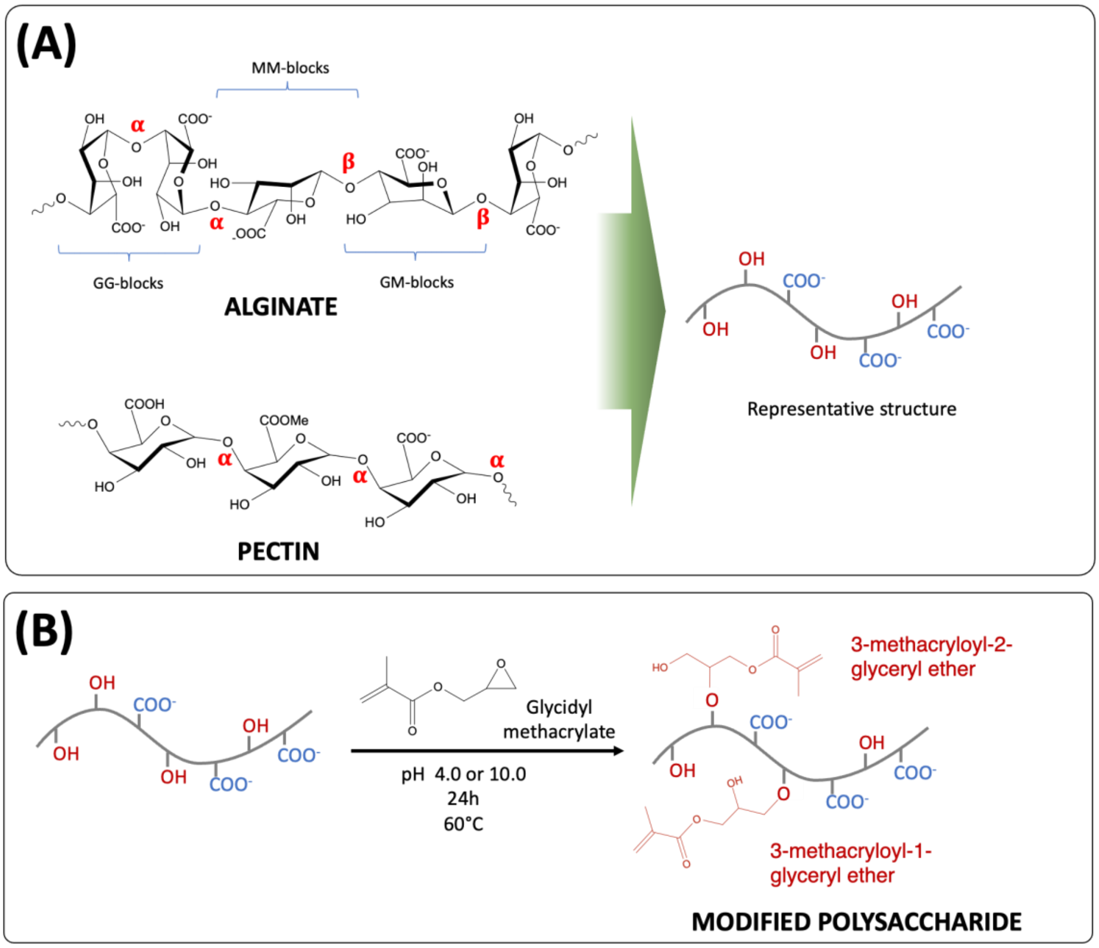

Figure S1. Schematic representation of regioisomeric products of the modified polysaccharide.

S2. Integration of Eq. (5):

Eq. (5) is a first-order linear differential equation, which can be rewritten as follows:

$$\frac{dw_{water}(t)}{dt} + kw_{water}(t) = kw_{water}^{max}$$

After multiplying the above equation by e^{kt} (integrating factor), the following expression is obtained:

$$\frac{dw_{water}(t)}{dt}e^{kt} + kw_{water}(t)e^{kt} = kw_{water}^{max}e^{kt}$$

which can be rearranged as:

$$\frac{d}{dt}[w_{water}(t)e^{kt}] = kw_{water}^{max}e^{kt}$$

by integrating this equation, we obtain:

$$\int d[w_{water}(t)e^{kt}] = kw_{water}^{max}\int e^{kt}dt$$

which gives Eq. (6):

$$w_{water}(t) = w_{water}^{max} + Ae^{-kt}$$

Substituting Eq. (6) in Eq. (3), we obtain Eq. (7):

$$w(t) = w_o + w_{water}^{max} + Ae^{-kt}$$

Finally, as $F(t) \propto w(t)$, and by applying the boundary condition ($t = 0$ implies $F(0) = 0$ – no water absorption), then:

$$F(t) = \alpha[w_o + w_{water}^{max} + Ae^{-kt}]$$

$$0 = \alpha[w_o + w_{water}^{max} + A]$$

$$A = -(w_o + w_{water}^{max})$$

substituting A in $F(t)$ and making $w_{eq} = w_o + w_{water}^{max}$, we obtain the Eq. (8).

S3. The random walk problem

Consider two crosslinking points initially separated by $\vec{l_o}$. At a certain time δ, one of them will move in a length l_i randomly, as illustrated in the Figure below:

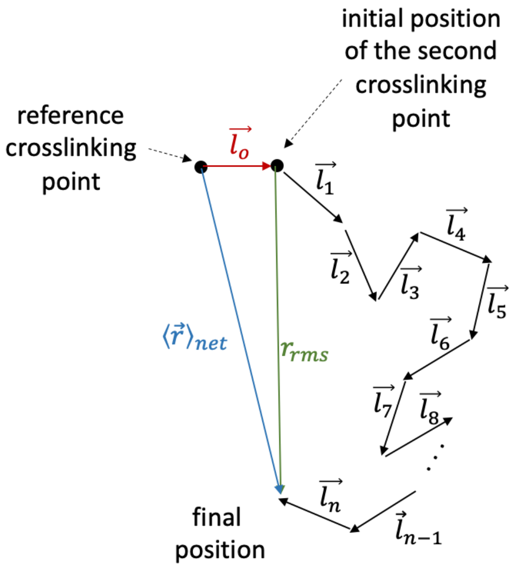

Figure S2. A representation of the random movement between two crosslinking points.

After n steps, the average distance traveled ($\langle\vec{r}\rangle_{net}$) by the crosslinking point will be given by the vectorial sum of all vectors $\vec{l_\iota}$, while the total time t of this movement will be given by $n\delta$.

A better way to describe $\langle\vec{r}\rangle_{net}$ is considering that $\langle\vec{r}\rangle_{net} = \vec{l_o} + r_{rms}$, where r_{rms} is the root mean square of the relative distance traveled by the crosslinking point as a function of time. The r_{rms} is defined as:

$$r_{rms} = \langle\vec{r}\cdot\vec{r}\rangle^{1/2}$$

where the term $\langle\vec{r}\cdot\vec{r}\rangle$ is the mean square of the distance given by:

$$\langle\vec{r}\cdot\vec{r}\rangle = \sum_{i=1}^{n}\vec{l_\iota}\cdot\sum_{j=1}^{n}\vec{l_J}$$

The sum of the above equation can be separated into a set of sums:

$$\langle\vec{r}\cdot\vec{r}\rangle = \sum_{i=j}^{n}\vec{l_\iota}\,\vec{l_J} + \sum_{i\neq j}^{n}\vec{l_\iota}\,\vec{l_J}$$

The term with *i≠j* is equal to zero since for each pair $\vec{l_i}\,\vec{l_j}$ there will be another with opposite signal. On the other hand, the term with *i=j* will be approximately equal to nl^2, since all vectors $\vec{l_i}$ have approximately the same length l. Thus, the r_{rms} for a single crosslinking point will be:

$$r_{rms} \approx l\sqrt{n}$$

at a certain time t, the above equation can be written as follows:

$$r_{rms} \approx l\left(\frac{t}{\delta}\right)^{1/2}$$

Thus, the temporal dependence of $\langle r \rangle_{net}$ is given by:

$$\langle r \rangle_{net} \approx \vec{l}_o + l\left(\frac{t}{\delta}\right)^{1/2}$$

As mentioned in the *section 3.1.3*, we can conclude that $\langle r \rangle_{net} \propto t^{1/2}$.

S4. Derivation of the temporal dependence of ρ^{net}

Considering that ρ^{net} is proportional to $d\langle r\rangle_{net}/dt$, thus:

$$\rho^{net} \propto \frac{1}{t^{-1/2}}$$

Assuming that this proportionally has a linear behavior, we will obtain:

$$\rho^{net} = \frac{\beta}{t^{1/2}} + \kappa$$

where β and κ are proportional constants. Then, by applying the boundary condition that at $t \to \infty$, $\rho^{net} \to \rho_{eq}^{net}$ we can find that $\kappa = \rho_{eq}^{net}$. Finally, substituting ρ_{eq}^{net} in the above equation, we can obtain Eq. (10).

S5. The contribution of Fickian diffusion

The Eq. (20) can be rewritten as:

$$S \approx \overbrace{\frac{a^*}{t^{1/2}}\left(\sum_{n=1}^{\infty}\frac{(-1)^{n+1}k^n t^n}{n!}\right)}^{f} + \overbrace{b^*\left(\sum_{n=1}^{\infty}\frac{(-1)^{n+1}k^n t^n}{n!}\right)}^{r}$$

Thus, the relation r/f will be equal to $b^* t^{1/2}/a^*$.

The Eq. (21) can be rewritten as:

$$CF = \frac{1}{1+\frac{r}{f}}$$

Finally, substituting r/f we will obtain the Eq. (22).
